# Multi-Facial, Non-Peptidic α-Helix Mimetics

**DOI:** 10.3390/biology4030540

**Published:** 2015-08-31

**Authors:** Maryanna E. Lanning, Steven Fletcher

**Affiliations:** 1 Department of Pharmaceutical Sciences, University of Maryland School of Pharmacy, 20 N. Pine St., Baltimore, MD 21201, USA; E-Mail: mlanning@umaryland.edu; 2 University of Maryland Greenebaum Cancer Center, 22 S. Greene St., Baltimore, MD 21201, USA

**Keywords:** α-helix mimetic, protein–protein interaction, proteomimetic, amphipathic, cancer, Bcl-2, HMD2, Bak, Bim, p53

## Abstract

α-Helices often recognize their target proteins at protein–protein interfaces through more than one recognition face. This review describes the state-of-the-art in the design of non-peptidic α-helix mimetics that reproduce functionality from multiple faces of an α-helix.

## 1. Introduction

Protein–protein interactions (PPIs) are involved in an array of crucial cellular processes that include differentiation, apoptosis, signal transduction and transcription [1,2,3]. Thus, their dysregulations render them attractive therapeutic targets for the treatment of a variety of diseases, including cancer, diabetes, neurodegenerative disorders and HIV/AIDS [4,5,6,7,8]. Long considered an insurmountable challenge, the scientific literature is now rife with synthetic strategies to disrupt aberrant PPIs, and, whilst the number of such small-molecules that have reached the clinic remains low, e.g., ABT-737/263, the development of chemical probes *en* *route* to drug candidates has assisted in the delineation of PPI-mediated cellular processes [9,10]. PPIs boast solvent-exposed, large, flat and predominantly hydrophobic interfacial areas (often, as much as 1600 Å^2^ is buried but this can rise to 4500 Å^2^) that comprise non-contiguous contact points, indicating that large, hydrophobic molecules might be mandatory for effective disruption [1]. These features are in stark contrast to enzyme active sites where small-molecules can be fashioned after the enzyme’s substrate or designed *de novo* to recognize the well-defined cavity. Furthermore, hydrophilic groups can be more readily exploited, which directly aids in compound solubility.

Despite the large interfacial areas involved in PPIs, much of the free energy of binding is attributed to “hot spots”, smaller domains within the PPI that are more tractable to low-molecular-weight ligand design [11]. One such subset of PPIs is described by those that are mediated by α-helices, wherein key residues projected from the helix recognize hot spots in the target protein [12,13,14]. α-Helices are found at the interfaces of almost two-thirds of the PPIs found in the Protein Data Bank (PDB), demonstrating their significance in protein–protein recognition, and around half of these involve the helix utilizing a single recognition face [12]. Broadly speaking, synthetic strategies to disrupt helix-mediated PPIs fall into two categories: peptidic and non-peptidic scaffolds. In the former category, Cabezas [13] and Arora [14] have independently enforced helical conformations of peptides by replacing internal hydrogen bonds with covalent linkages; this approach has led to the discovery of a novel inhibitor of the HIF1α–p300 PPI [15]. Meanwhile, as a follow-up to Grubbs’s earlier report that a ring-closing metathesis reaction between two unnatural amino acids carrying alkenyl-based side chains promoted helicity, Verdine later demonstrated that such “stapled” peptides can inhibit the Bid–Bcl-2 PPI, amongst others, and, despite the highly peptidic nature of the inhibitor, was biologically active *in* *vivo*, reducing the growth of human leukemia xenografts [16,17]. Other chemical conformational constraints between amino acid side chains include lactams [18], di-sulfide bridges [19], intramolecular salts [20], and triazoles [21]. A third peptidic strategy to accomplish helix mimicry takes advantage of the helical propensity of β-peptides first shown by Gellman, Seebach and co-workers, which has resulted in the disruption of the p53–MDM2 and Bim–Bcl-2 PPIs [22,23].

**Figure 1:**
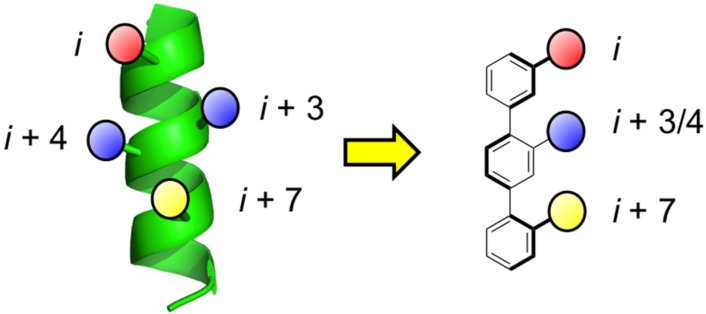
An α-helix (**left**) and a terphenyl-based α-helix mimetic (**right**), highlighting the amino acid side chains located on one face. Colours correspond to amino acid side chains and their respective surrogates.

**Figure 2:**
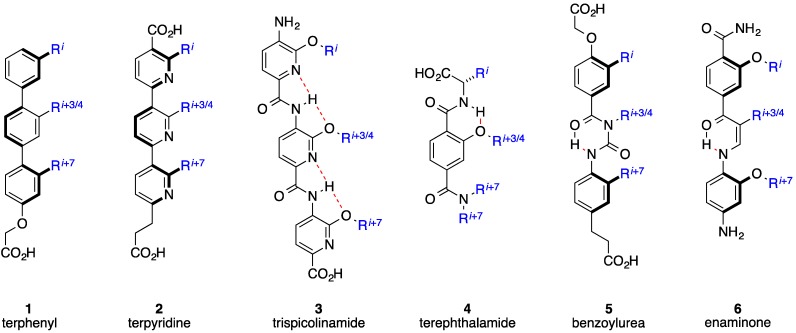
Single-faced, synthetic α-helix mimetics. Dashed lines represent hydrogen bonds.

**Figure 3:**
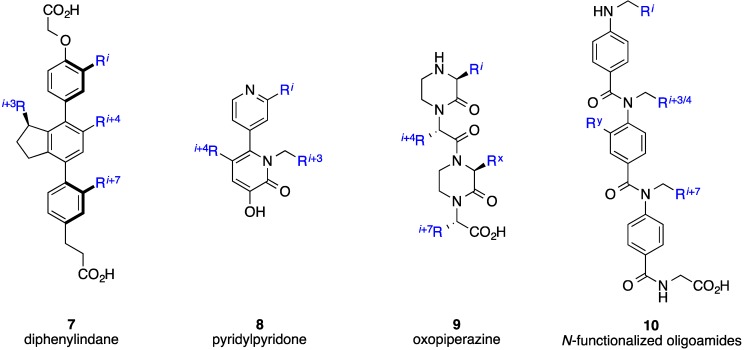
Multi-sided, single-faced, synthetic α-helix mimetics. R^x^ and R^y^ may be solubilizing groups or moieties to improve cell uptake, for example.

Some drawbacks of the peptidic strategy to mimic α-helices include potential metabolic instability through digestion of peptide bonds, as well as limited cell penetrating properties due to the presence of charged side chains and the cumulative effect of the polar peptide bonds. The second category of helix mimetics encompasses structures of non-peptidic design, and so aims to address the limitations of the previous category. Synthetic, non-peptidic agents that can accurately project functional groups from a scaffold in an orientation similar to the native α-helix have effectively disrupted helix-mediated PPIs within cells [24,25]. Although the first report on helix mimicry with synthetic agents was published by Howson and co-workers [26,27], it was Hamilton’s laboratory that pioneered the field of helix mimicry with low-molecular-weight ligands covering a wide range of synthetic scaffolds including terphenyls [28,29], terpyridines [30], trispicolinamides [31], terephthalamides [32,33], benzoylureas [34], and enaminones [35,36]. Since many α-helices utilize only one face to recognize their partner proteins, specifically through the side chains of the *i*, *i* + 3/4 and *i* + 7 residues, the development of helix surrogates has traditionally focused on the mimicry of these residues and hence on only one face of an α-helix; as an example, a schematic of an archetypal terphenyl helix mimetic alongside an α-helix is given in Figure 1, wherein the *ortho* substituents are designed to mimic the *i*, *i* + 3/4 and *i* + 7 residues. In addition to excellent reproduction of the relative distances between the side chain mimics, it is important to note that the subunits within the terphenyls are staggered due to steric interactions, which further promotes helix mimicry since the *i*, *i* + 3/4 and *i* + 7 residues are likewise staggered. Several of the different types of scaffolds that have been introduced as single-faced helix mimetics are illustrated in Figure 2 and are reviewed in more detail elsewhere [37,38,39,40,41,42]. Additionally, a range of multi-sided helix mimetics that are intended to mimic only one face of an α-helix, including oxopiperazines and *N*-functionalized oligoamides, are given in Figure 3 [43,44,45,46,47]. The R^3^ group of the oxopiperazines was hypothesized in the general case to allow for the introduction of a solubilizing group, but might also reasonably mimic the *i* + 3 side chain, providing the reproduction of four side chains on one face of a helical epitope [43]. Interestingly, although the *N*-functionalized oligoamides were introduced as a means to incorporate groups opposite the helix-mimicking epitope for cell studies and to promote cellular uptake, one may envisage this scaffold also being developed into a platform for multi-facial α-helix mimicry [44]. Klussmann and colleagues have described synthetic chemistry to achieve highly-substituted terpyridines and demonstrated the disruption of the protein kinase A (PKA)–A-kinase anchoring protein (AKAP) through effective mimicry of the helical domain of AKAP. This also opens the door for utilizing the terpyridine scaffold to realize multi-facial α-helix mimicry [48]. Increasingly, two- and three-sided helix-mediated PPIs are being discovered at therapeutically important interfaces [12]. Many of the conformationally flexible Hamilton and Hamilton-type scaffolds, such as the pioneering terphenyls, that were designed to mimic the residues on only one face of an α-helix may be capable of mimicking residues on multiple faces by virtue of unrestricted rotation about an aryl–aryl bond, for example. That being said, since preorganization is considered to enhance the free energy of binding owing to a reduced entropic penalty, the focus of this review is multi-sided helix mimetics that, through covalent and/or non-covalent bonds, are preorganized to reproduce functionality on multiple faces, in contrast to the those structures in Figure 3 that are designed to mimic only one face of an α-helix.

## 2. Bis-Benzamides

**Figure 4:**
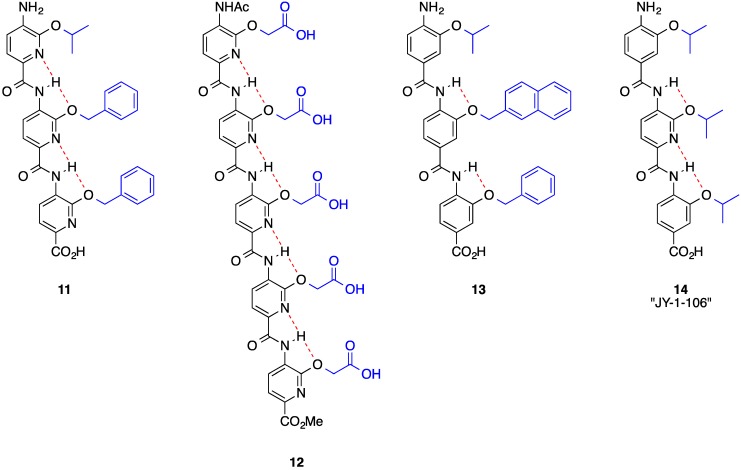
Oligoarylamides as single-faced helix mimetics.

An alternative and synthetically more accessible helix mimetic to the terphenyls are the tris-picolinamides [31], which have formed the basis for two-faced helix mimicry (see below). However, given their rich contribution to single-faced helix mimicry, we first begin with a summary of the simpler oligoarylamides. Through mimicry of the *i* (V74), *i* + 4 (L78) and *i* + 7 (L81) side chains of the Bak-BH3 α-helix with alkoxy groups, representative trispicolinamide **11** (Figure 4) disrupted the Bak–Bcl-x_L_ PPI with a *K*_i_ of 1.6 μM. The anti-apoptotic Bcl-2 proteins, which include Bcl-x_L_, Bcl-2 and Mcl-1, neutralize the pro-apoptotic Bcl-2 proteins, such as Bak and Bim, by capturing their BH3 α-helical “death domains” [4]. Since the upregulation of the anti-apoptotic Bcl-2 proteins has been associated with the development and progression of various cancers [4], compound **11** provides a starting point from which new antineoplastics might be realized. The role of the pyridine nitrogens was to promote the projection of the three side chains from the same face of the mimetic through bifurcated hydrogen bonds. Oligomerization of the picolinamide core generated an inhibitor of islet amyloid polypeptide aggregation (compound **12**, Figure 4) [49,50], whilst Wilson replaced the pyridine rings with benzene rings, which afforded potent inhibitors, such as **13**, of the p53–hDM2 PPI through accurate mimicry of F19 (*i*), W23 (*i* + 4), L26 (*i* + 7) of the p53 helix [51]. The therapeutic significance of Wilson’s work lies in the potential discovery of new anti-cancer drugs since the tumor suppressor role of p53 is inactivated by hDM2 [52]. Using the Bak–Bcl-x_L_ PPI as a tool, the Fletcher group interrogated the role of the preorganizing pyridine nitrogen atoms of a trispiconlinamide through systematically replacing the pyridine nitrogens with CH groups (*i.e.*, benzene rings), resulting in the potent and cell-active compound **14** (JY-1-106) [25,53].

**Figure 5:**
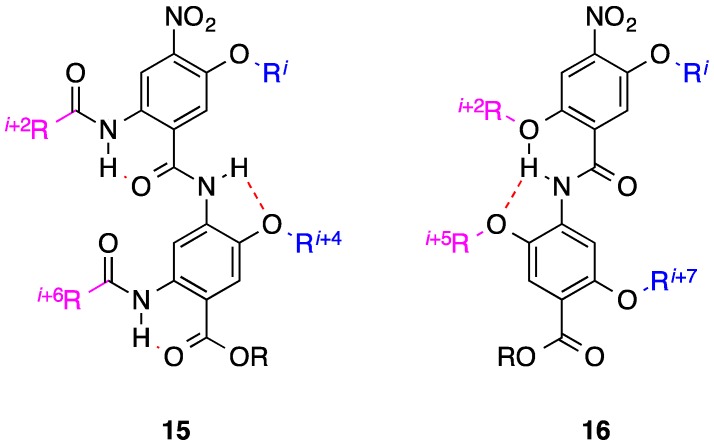
Two-faced bis-benzamide helix mimetics. Dashed lines represent hydrogen bonds.

As alluded to earlier, the oligoamides, which present simpler syntheses than their terphenyl predecessors, are attractive candidates for accomplishing the mimicry of two faces of an α-helix. Accordingly, two groups independently converted single-faced bis-benzamides into two-faced helix mimetics. Hamilton’s laboratory introduced amides opposite the alkoxy-decorated face to allow for the additional mimicry of the *i* + 2 and *i* + 6 side chains; the generic structure is illustrated by compound **15** (Figure 5) [54]. The desired conformational constraints hinged on the formation of six-membered intramolecular hydrogen bonds, which were confirmed by X-ray crystallography. In a complementary strategy, Anh’s group developed synthetic chemistry to introduce additional alkoxy functionality into the opposing face that, through a bifurcated hydrogen bond, also permits mimicry of two opposing recognition faces of an α-helix (compound **16**, Figure 5) [55]. A crystal structure revealed five- and six-membered, intramolecular hydrogen bonds between the amide NH and the ether oxygens, resulting in excellent spatial mimicry of the *i*, *i* + 7 and *i* + 2, *i* + 5 side chains from opposite faces. To the best of our knowledge, no biological data has been reported on these new, multi-facial bis-benzamides. It is anticipated that both types of bis-benzamide will allow the introduction of hydrophobic and hydrophilic groups.

## 3. Benzoylureas

**Figure 6:**
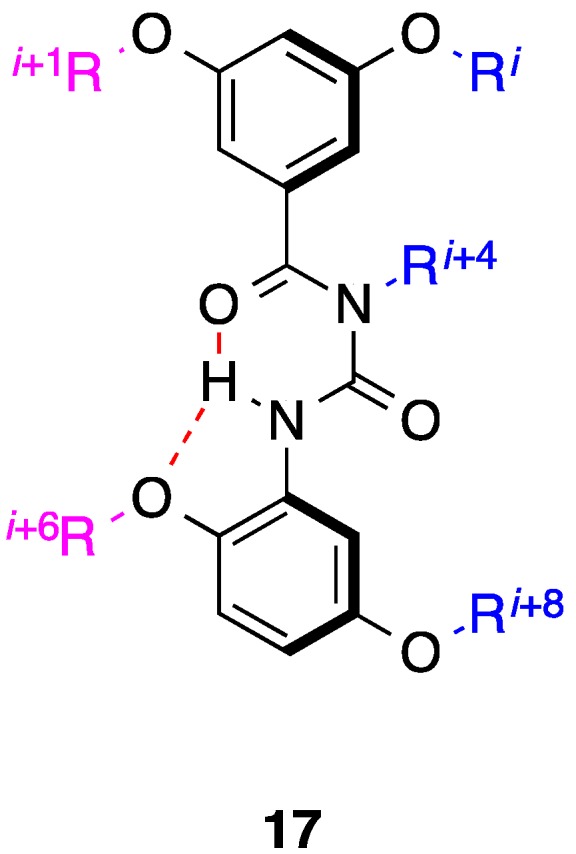
A two-faced helix mimetic centered on a benzoylurea scaffold. Dashed lines represent a bifurcated hydrogen bond.

Hamilton demonstrated that five residues (*i*, *i* + 1, *i* + 4, *i* + 6 and *i* + 8) spanning two turns and opposing faces of an α-helix could be effectively reproduced through the elaboration of a benzoylurea scaffold that was previously reported by the group [34,56]. Confirmation that the two-faced benzoylurea **17** (Figure 6) folds into a helical-type structure through a bifurcated hydrogen bond was provided by X-ray crystallography, although whether this organized structure can be attained under physiological conditions is unknown. Unlike the bis-benzamides, the “upper” phenyl ring of the benzoylureas is not subjected to non-covalent interactions, providing a two-faced helix mimetic with greater conformational flexibility, as well as the ability to emulate a fifth side chain.

## 4. 1,2-Diphenylacetylenes

**Figure 7:**
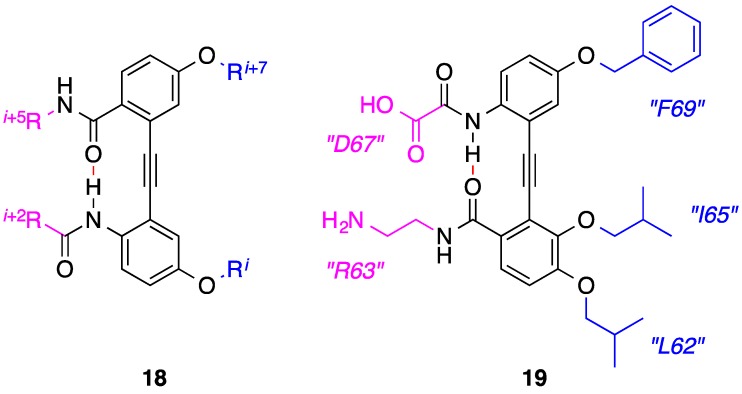
An intramolecular hydrogen bond (dashed line) influences the projection of side-chains from opposing faces of a diphenylacetylene scaffold.

Functionalized 1,2-diphenylacetylenes have recapitulated the epitopes of β-turns and β-strands by virtue of strategically fashioned intramolecular hydrogen bonds [57,58,59,60]. The Fletcher group considered that this framework might also be exploited to elicit two-faced α-helix mimicry [61]. The prototypical compound **18** (Figure 7) was prepared, which, according to computational modeling, reproduced the spatial and angular projections of the *i* and *i* + 7 side chains on one face and the *i* + 2 and *i* + 5 on the opposite face. The presence of the key intramolecular hydrogen bond to influence the projection of specific side chains from opposing faces of the scaffold was then ratified in the solution state by ^1^H NMR titration experiments. As stated earlier, the oncoprotein Mcl-1 neutralizes the pro-apoptotic Bcl-2 proteins, which include Bim and Bak, through binding their BH3 domains. Elaboration of the design, as in the amphipathic helix mimetic **19**, allowed for the mimicry of five residues of the Bim-BH3 α-helix located across two faces and within two turns of the helix: *i*, *i* + 1, *i* + 3, *i* + 5 and *i* + 7. In addition to structurally mimicking the Bim-BH3 α-helix, the group demonstrated that **19** was also a functional helix mimetic, inhibiting Mcl-1 with a *K*_i_ of 3.24 μM, as determined by a fluorescence polarization competition (FPC) assay.

## 5. Anthraquinones and Acridines

**Figure 8:**
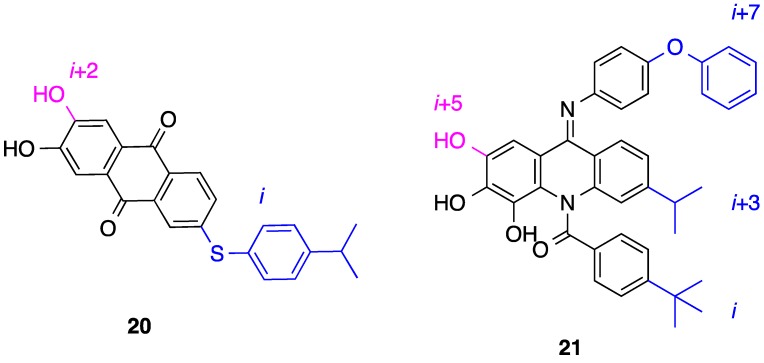
Appropriately functionalized anthraquinones (**left**) and acridines (**right**) have disrupted the Bim–Mcl-1 PPI through two-faced mimicry of the Bim-BH3 α-helix.

Zhang and colleagues designed and evaluated anthraquinones as two-faced helix mimetics wherein substitution of opposite sides of the rigid scaffold affords reasonable mimicry of side chains located on opposing faces of an α-helix [62]. The width of the anthraquinone core (4.8 Å) is similar to the diameter of an α-helix, and so it was postulated that appropriate functionalization of the two edges of the scaffold would allow for mimicry of D67 and I65 of the Bim-BH3 α-helix. Pleasingly, the group identified nanomolar dual inhibitors of the Bim–Mcl-1 and Bim–Bcl-2 PPIs, with their most potent compound, **20** (Figure 8), exhibiting *K*_i_’s of 13 and 24 nM for Mcl-1 and Bcl-2, respectively, according to FPC assays. HSQC NMR experiments with **20** resulted in significant chemical shift perturbations of many residues in the hydrophobic groove of ^15^*N*-labeled Mcl-1, most particularly those of R263/N260 and V220/H224/V274/F228, corresponding to hotspots recognized by D67 and I65 of the Bim-BH3 α-helix, respectively. Taken together, these data suggest anthraquinone **20** inhibits Mcl-1 and Bcl-2 through structural and functional mimicry of opposing faces of the Bim-BH3 α-helix. The same group developed their anthraquinone into an elaborate acridine scaffold that could accomplish additional mimicry of two more residues [63]. Acridine **21** bound Mcl-1 and Bcl-2 with *K*_i_ values of 79 and 56 nM, respectively, through proposed mimicry of the *i*, *i* + 3, *i* + 5 and *i* + 7 side chains of the Bim-BH3 α-helix. Once again, HSQC NMR spectroscopy corroborated Zhang’s design, indicating that **21** accurately mimicked the side chains of L62, I65, F69 and D67 of Bim. Direct binding of **21** to Mcl-1 was confirmed by isothermal titration calorimetry (ITC) (*K*_d_ = 107 nM), substantiating the affinity determined in the FP assay. Finally, since some acridines are DNA intercalators, **21** was evaluated for its effect on DNA: no significant differences in superhelical content or band shape in agarose gel electrophoresis was detected indicating this acridine does not interact with DNA.

## 6. 2,6,9-Tri-Substituted Purines

**Figure 9:**
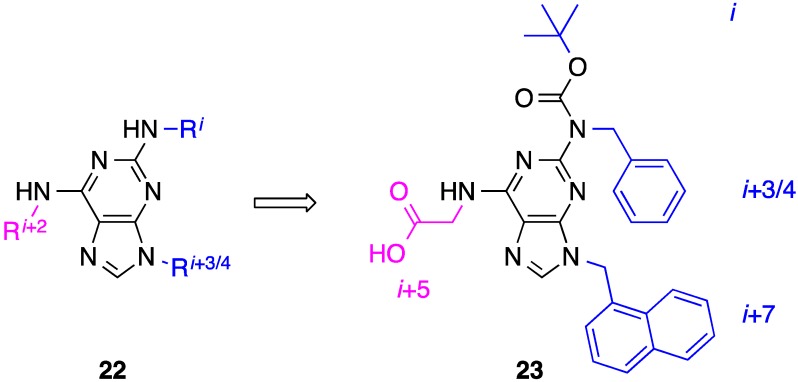
2,6,9-Tri-substitution of a purine scaffold permits the mimicry of two faces of an α-helix, according to the disruption of the Mcl-1–Bak-BH3 PPI.

Towards the disruption of the p53–MDM2 PPI, the Lim group recently developed single-faced helix mimetics of the p53 α-helix, based on a pyrrolopyrimidine core, and, despite its rigidity, several potent inhibitors of MDM2 were discovered [64]. Their research thus highlighted that a flexible scaffold for helix mimicry is not crucial, presumably provided the side chains are sufficiently flexible that mimicry of staggered side chains can be accomplished. Inspired by this work, and having previously developed synthetic chemistry to control the N9-regioselective alkylation of the purine nucleus [65], the Fletcher group introduced 2,6,9-tri-substituted purines as two-faced helix mimetics [66]. On the simplest level, this scaffold was proposed to mimic the *i*, *i* + 2 and *i* + 3/4 side chains across opposing faces within one turn (compound **22**, Figure 9). Elaboration of their design allowed for the additional mimicry of a fourth residue: *i*, *i* + 3/4, *i* + 5 and *i* + 7, corresponding to L78, I81, D83 and I85 of the Bak-BH3 α-helix. A small library of compounds was prepared, the most potent member of which, **23**, disrupted the Bak-BH3–Mcl-1 PPI with an estimated IC_50_ of 72 μM. It was hypothesized that the rigid *tert*-butyl of the Boc group would be a poor mimetic of the *i* side chain, and this may account for the moderate inhibition, but was incorporated into the design to facilitate the synthetic chemistry and allow the rapid evaluation of such functionalized purines as helix surrogates. Thus, to address this caveat, the group is presently focusing on the replacement of the Boc group with more flexible carbamates and sulfonamides to realize better helix mimicry.

## 7. Tetrahydro-1H-benzo[*e*][1,4]diazepines

**Figure 10:**
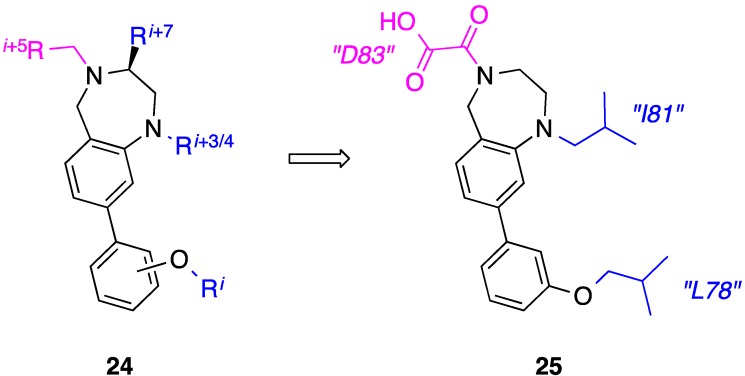
In addition to their ability to mimic the epitopes of β-turns and one face of an α-helix, benzodiazepines have been introduced as scaffolds to reproduce functionality displayed from two faces of an α-helix.

Benzodiazepines have a rich and privileged history in medicinal chemistry, inclduing in proteomimicry, where they have featured as scaffolds for the mimicry of β-turns and one face of an α-helix [67,68]. Due to the rigid framework and projection of substitutable nitrogen atoms from opposite sides, the Fletcher group considered that tetrahydro-1*H*-benzo[*e*][1,4]diazepines (BZDs) could also lend itself to two-faced helix mimicry, reproducing the *i*, *i* + 3/4 and *i* + 7 side chains on one face, and the *i* + 5 residue on the other (generic structure **24**, Figure 10) [69]. A small library of appropriately functionalized BZDs was prepared and then assessed for their abilities to disrupt the Bak-BH3–Mcl-1 PPI. The most potent compound was the amphipathic helix mimetic **25**, which inhibited Mcl-1 with a *K*_i_ = 6.67 μM. The group is currently streamlining the synthetic chemistry to facilitate the assembly of a large BZD library to further interrogate the Bak-BH3–Mcl-1 PPI, as well as other helix-mediated PPIs.

## 8. Phenyl-Piperazine-Triazine

**Figure 11:**
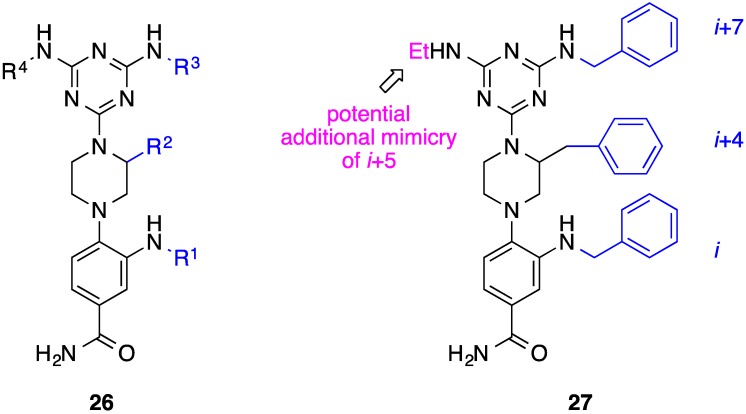
A phenyl-piperazine-triazine helix mimetic disrupts the Bim–Mcl-1 PPI. Synthetic strategies to vary the ethyl group should allow the additional mimicry of the *i* + 5 position on the opposing face.

Towards improving the physicochemical properties of traditional helix mimetics, Lim and co-workers devised a unique helix mimetic constructed from a phenyl-piperazine-triazine core (generic structure **26**) to target the Bim-BH3 –Mcl-1 PPI [70]. The authors exploited solid phase chemistry to accomplish rapid diversification, creating a library of 36 compounds. For prototypical compounds in which all three side chains are methyls, the phenyl-piperazine-triazine helix surrogate sports a cLog *P* of 0.96, which contrasts considerably with the analagous terphenyl scaffold (cLog *P* = 6.02). The library members were tested for their abilities to inhibit Mcl-1 using an FPC assay; their most potent compound, **27** (Figure 11), with R^1^ = R^2^ = R^3^ = Bn, had a *K*_i_ of 7.3 μM based on the FP data. Lim’s group also evaluated the specificity for Mcl-1 over the related anti-apoptotic Bcl-2 protein Bcl-x_L_, both of which have similar BH3-binding grooves [70]. Interestingly, **27** exhibited no affinity to Bcl-x_L_ up to 100 μM, indicating this new helix mimetic is capable of discriminating between the two Bcl-2 proteins. It is unclear if the Mcl-1 selectivity can be attributed to the specific side chains or the scaffold itself since only one library member was evaluated in the selectivity assay. Retrosynthetic analysis of the triazine moiety reveals its origin to be cyanuric trichloride, which may be triply-functionalized, allowing side chains on opposing faces of an α-helix to be emulated. Thus, although this scaffold presently has been used to realize the imitation of only one face of an α-helix, Lim’s work inherently provides a platform to achieve two-faced helix mimicry (*i* + 5 position), which was hinted at in their abstract. Finally, the stereogenic center in the piperazine ring will permit the investigation into the effect of helix mimetic chirality on protein recognition.

## 9. Azabicyclo[2.2.2]Octane Aryl Amide

**Figure 12:**
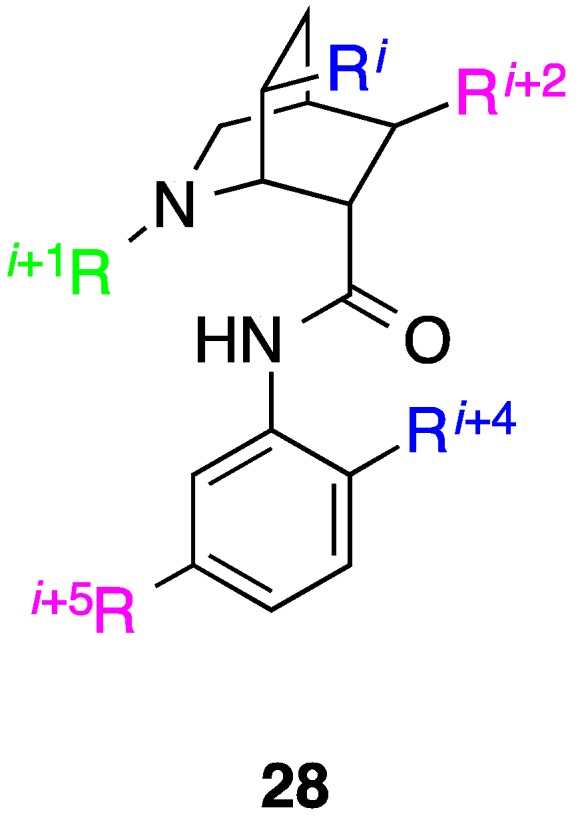
A novel helix mimetic that projects functionality in a similar orientation to five of seven residues spanning two turns of an α-helix.

The two-faced bis-benzamides and benzoylureas described thus far were developed from single-faced variants and owing to the planarity of the benzene rings, mimicry of additional residues on another faces is restricted to those that lie 180º to the existing side chains. In a *de* *novo* approach, Spivey and co-workers designed and synthesized an azabicyclo[2.2.2]octane aryl amide scaffold that is capable of mimicking five residues: *i*, *i* + 1, *i* + 2, *i* + 4 and *i* + 5 [71]. With the prototype compound **28** (Figure 12) in hand where all side chains are methyls, the group is currently optimizing the synthetic chemistry to facilitate the generation of an array of azabicyclo[2.2.2]octane that will be decorated with hydrophobic groups to afford therapeutically useful helix mimetics.

## 10. Conclusions

The mimicry of one face of an α-helix with non-peptidic scaffolds is now a well-established field of research, although clinical candidates to disrupt aberrant helix-mediated PPIs remain elusive. Since α-helices often recognize their target proteins through multiple recognition faces, it is considered that the reproduction of functionality from multiple faces of an α-helix might afford more potent and more selective agents. In turn, it is reasoned that such multi-facial helix mimetics might result in the discovery of potential therapeutic agents suitable for advancement into preclinical development. Synthetic scaffolds that accomplish the mimicry of opposing faces of an α-helix are beginning to emerge, and it is hoped that enthusiasm in this area of research will intensify in future years to expand the drug armamentarium in the battle against pathogenic PPIs.
